# Extract from the Minutes of the Atlanta Academy of Medicine

**Published:** 1871-10

**Authors:** 


### EXTRACT FROM THE MINUTES OF THE ATLANTA ACADEMY OF MEDICINE.

REPORTED BY WM. ABRAM LOVE, M.D., CORRESPONDING SECRETARY.

August 25th, 1871.

Discussion of medical subjects being in order, Dr. V. H. Taliaferro, of Columbus, Georgia, being present, was called on to give the Academy the benefit of his experience in the use of his "*cloth tents*," specimens of which had been presented to the Academy on a former occasion.

Dr. Taliaferro stated that, for dilatation, they were not originally intended to supply the place of, or supplant the *sponge* or Laminaria; but as therapeutic agents, he thought them far preferable, as there did not exist the same objections that were found to obtain in the others. In his experience, he had found the sponge and sea-tangle produce too much irritation.

From the cloth tents, he had found all the good results, without the evils. And again: they could be medicated with facility to meet the indications in any particular case or condition.

For *dilating*, he had found their continued use—increasing the size from day to day—produce such an amount of dilation as might be desired, without the irritation following the other tents, especially where there exists a condition of disease in the mucous surface.

Again: the cloth tent will retain more of any one particular medical agent than can be introduced in or through the sponge or sea-tangle. By gradual absorption, it will take up the desired quantity of iodine, or other substances; and in this way, the internal surface of the uterus can be more effectually medicated than by the same substance used for or by injection.

Dr. Taliaferro states that he had formerly used medicines by injection after dilating the os and neck; but experience has taught him that he can medicate more effectually with the cloth tent, from the fact that the agent used is brought in contact with the diseased surface, or tissue, more effectually, more slowly, more continuously, and, at the same time, more prominently. While, on the one hand, injections merely coat the mucous membrane with the substance used, the medicated tent remains, say twenty-four hours, in continuous contact, the remedies are diffused over the tissues, absorption is certain and effectual, the whole surface being reached by the long-continued contact. These tents, medicated with iodine, or iodine and bromine, or glycerine, he has found very serviceable in irritable uterus.

Having soaked the tent for several days, it is then carried well into the cavity of the uterus, and allowed to remain. An astonishingly large quantity of fluid can, in this way, be drained from the organ, which reduces the engorgement and redness, and overcomes the irritability. In this way, he has used tanno-glycerine, sulphate of zinc, chromic acid, nitrate of silver, etc., introduced and withdrawn within five minutes, according to the impression desired to be made.

The tent is light; it may be curved to suit the uterine cavity, and is easily made; it is cheap, and the material is always at hand.

Dr. Taliaferro *demonstrated* the mode of making cloth tents: A strip of cloth of the desired width is rolled firmly between the finger and thumb, regulating the size and length by distance of lap in the roll and length of cloth rolled into one tent, the strips being about half an inch wide.

Dr. J. F. Alexander inquired, how that tent could apply medicine to the whole of the internal surface of the womb?

Dr. Taliaferro replied, by diffusion.

Dr. Cumming.—How can that apply the remedy to all the surface?

Dr. Taliaferro.—If necessary, use larger tents—dilate, and use larger tents; but the remedy will diffuse itself over the surface. Should the agent used prove too irritating, producing abrasion of the surface, with a more mild medication, this objection will not obtain.

Dr. Taliaferro preferred this tent to Peasley's fenestrated tubes, for the reason that he could not use them, for the special local application, to a point, as was claimed for them.

Dr. W. F. Westmoreland asked, how Dr. Taliaferro would medicate with iodine?

Dr. Taliaferro.—Dip the tent into Lugol's solution, or tincture of iodine—let it dry or not; or use ointment. The tent, once charged thus, will not empty itself *in utero* in less than twenty-four hours—during which time the patients often complain of the taste of the medicine used endometrically.

Dr. Wells thinks the point of the tent has not sufficient firmness to enter the internal os.

Dr. Taliaferro.—Has never any difficulty in their introduction; they will not double or bend, as supposed.

Dr. Logan stated that he had used one, which passed to the fundus with the greatest facility.

Dr. Taliaferro stated, that another advantage in the tent was the facility with which they could be removed: by the string attached, they could be removed at any moment by the patient, which could not be done with the sponge, by reason of its distension and becoming imbedded in the mucous membrane.

Dr. Taliaferro states, that when the tent is removed, he washes out the cavity of the uterus; the tent so paralyzes the internal os as to prevent its contraction on using the wash. If the os has not been sufficiently dilated for washing out the cavity, he inserts a larger tent, until the requisite distension is produced, when he washes it out—with salt and water usually. The cloth of which the tents are made should not contain starch, for the reason that it is incompatible with iodine, etc. He sometimes continues the use of a succession of tents for a week or more, to reduce irritability of the uterus, using glycerine mainly for the purpose of draining off the engorgement.

Dr. Wells.—Will Dr. Taliaferro tell us if he has been satisfied with the results of his experience in the use of these cloth tents?

Dr. Taliaferro.—Certainly. I have; and believe this plan of treatment will cure more cases of endometritis than any other plan I have ever tried—with the tent made of ordinary bleached cloth, and medicated according to the indications to be met in each case.

Dr. W. F. Westmoreland.—Would like to know what has been Dr. Taliaferro's experience with injections in producing uterine colic, and such other accidents?

Dr. Taliaferro.—Has never had many accidents of this kind to contend with. Has always dilated well before using injections. He once used nitrate of silver, which was followed by colic. At another time, when half a drachm of tincture of iodine had been thrown in and syringe removed, contractions followed, with severe pain; in this case, he used a cloth tent, drained out the contents, and saved his patient. He believes that injections cause the internal os to close spasmodically, when contractions of the uterine walls may force the fluids used by injection through the Fallopian tubes, when this may be brought in contact with the peritoneum, and induce irritation, inflammation, etc., with all the evils that follow in such cases.

Dr. W. F. Westmoreland.—Thinks Dr. Taliaferro's opinion of the origin of uterine colic and nervous prostration correct: that is, when the internal os closes, the injection is forced into the Fallopian tubes; if it does not close, there is no pressure into the tubes. Dr. Taliaferro's paralysis of the internal os, by the tent, obviates this difficulty.

Dr. J. S. Wilson states, that his experience with injections is very limited. He injected warm water once (f ℥ i), which produced violent symptoms; this was his only experience. He thinks that something depends upon the condition of the womb, and that the internal uterus is very intolerant of foreign substances.

